# Comparison of two bone markers with growth evolution in 74 girls with central precocious puberty

**DOI:** 10.1186/s12887-018-1194-8

**Published:** 2018-07-09

**Authors:** Audrey Vincent, Jean-Claude Souberbielle, Raja Brauner

**Affiliations:** 1 Fondation Ophtalmologique Adolphe de Rothschild and Université Paris Descartes, 75940 Paris, France; 2 Hôpital Necker-Enfants Malades, Service d’Explorations Fonctionnelles, Assistance Publique-Hôpitaux de Paris, 75743 Paris, France

**Keywords:** Bone markers, Growth evolution, Central precocious puberty, Adult height, Bone alkaline phosphatase, CTX

## Abstract

**Background:**

The bone markers bone alkaline phosphatase (BAP) and C-terminal telopeptide of type I collagen crosslinks (CTX) are correlated with growth rate during normal puberty. The objective of this study was to evaluate the relationship between the serum concentrations of BAP and CTX and growth evolution in girls with idiopathic central precocious puberty (CPP) to help predict adult height.

**Methods:**

A retrospective single-center study was conducted in 74 girls with CPP for whom a serum sample at initial evaluation was available to retrospectively measure BAP and CTX concentrations; 66.2% of them were untreated.

**Results:**

The serum BAP concentrations showed significant positive correlations with height in standard deviations (SDS) at the initial evaluation (*n* = 62; *r* = 0.31; *p* = 0.015) and with the difference between bone and chronological ages (*n* = 61; *r* = 0.39; *p* = 0.002). BAP was also positively correlated with adult height as measured in both cm and SDS in untreated patients (*n* = 19; *r* = 0.58; *p* = 0.009).

The serum CTX concentrations showed significant positive correlations with growth rate the year before the initial evaluation as measured in both cm and SDS (*n* = 65; *r* = 0.34; *p* = 0.006).

**Conclusions:**

This study revealed significant correlations of serum BAP and CTX concentrations with growth evolution in girls with CPP. The high positive correlation between serum BAP and adult height in untreated girls suggests that BAP can possibly be used to optimize models of adult height prediction in girls with CPP.

## Background

Biochemical markers of bone turnover are routinely used in clinical practice to monitor osteoporosis in post-menopausal women [1]. In addition, such markers are being documented and utilized in children more frequently than in the past. Among these biochemical markers, bone alkaline phosphatase (BAP) is a marker of bone formation present in the membrane of the osteoblasts and released into the circulation. BAP is a more sensitive diagnostic tool than total alkaline phosphatase activity [2]. In contrast, C-terminal telopeptide of type I collagen crosslinks (CTX) is a marker of bone resorption that is generated from translational modification of bone collagen and is released during matrix resorption [2].

Some previous studies have evaluated these markers in healthy girls during puberty [3–7]. For example, it was demonstrated that BAP and CTX concentrations vary with chronological age [5, 6] and pubertal Tanner stage [3–6], which has led to established reference values [6, 7]. The peak levels of these bone markers occurred at Tanner breast stage 3 and decrease to adult concentrations at the end of puberty [3–6]. It has also been shown that BAP is positively correlated with growth rate (cm/year) during normal puberty [5].

Central precocious puberty (CPP) in girls is defined as the development of sexual characteristics before the age of 8 years due to premature activation of the hypothalamic-pituitary-ovarian axis [8]. In girls, CPP is idiopathic in the majority of cases [9]. Genetic [10, 11] and/or environmental factors, particularly obesity, contribute to early onset of puberty. An analysis of the presentation of 493 consecutive girls with CPP in a single-center study showed that obesity accelerates adrenarche but not maturation of the hypothalamic-pituitary-ovarian axis, which mainly depends on genetic factors [12].

Premature secretion of estradiol increases the growth rate and accelerates bone maturation, which can shorten the growing period, resulting in short adult height (AH). Treatment with a gonadotropin-releasing hormone (GnRH) analog blocks the pituitary-ovarian axis and thus estradiol secretion, thereby slowing bone age (BA) progression and preserving growth potential [13]. However, the effect of this treatment on AH varies, primarily because idiopathic CPP progression differs between unsustained forms, also called slowly progressing forms [14], and rapidly progressing forms [15].

Despite the breadth of reported data concerning AH in girls with idiopathic CPP, major questions remain about the indications for GnRH analog treatment [16]. As the reported height gain (AH-predicted AH at onset of treatment) varies from 0.3 [17] to 9.8 cm [18], it is difficult to determine whether to treat a given girl who has idiopathic CPP with GnRH analogs. Tools have been developed to predict AH in girls with CPP [19], but bone markers have not been used to date.

In this study, serum concentrations of BAP and CTX in girls with CPP were examined in relation to patient characteristics at initial evaluation, to growth rates at 6 months and 1 year after this evaluation, and to AH after spontaneous growth. The objective was to evaluate the relationship between serum concentrations of BAP and CTX and the growth evolution in girls with idiopathic CPP to help predict AH.

## Methods

### Study design

We conducted a single-center study in girls who had been monitored for idiopathic CPP by a senior pediatric endocrinologist (R. Brauner) in a university pediatric unit from June 1981 to July 2012 and for whom a serum sample at initial evaluation preserved at − 22 °C was still available to retrospectively measure serum BAP and CTX concentrations.

### Participants

All clinical investigations were conducted according to the principles outlined in the Declaration of Helsinki. Written informed consent for the evaluation was obtained from the children’s parents and included in their hospital medical record. With the exception of routine patient care, no other activities were performed for the study. The authors had no direct interaction with the patients enrolled in the study, except for their medical follow-up, which was performed by R. Brauner. Patients derived from a previous study [12] which was approved by the Ethical Review Committee (Comité de Protection des Personnes, Ile de France III) (Ref. CPP: AC 038), which states that “This study appears to be in accordance with scientific principles generally accepted and to ethical standards of research; this study was lead in the respect of the French law and regulation”.

The primary outcome was to evaluate the relationship between serum concentrations of BAP and CTX and the growth evolution, mainly the AH after spontaneous evolution. The secondary outcome was to compare these concentrations to the patient characteristics at initial evaluation.

The 74 girls included were selected from 493 girls with CPP [12]. At the initial evaluation, the characteristics of the 74 girls included in the study were similar to those of the 419 girls without available samples, except for basal FSH concentration and peak, which were significantly lower in the included patients (Table 1).

**Table 1:** Patients characteristics

			Patients included					Patients excluded				MWU
		value	*n*	Mean ± SD	Min	Max	%	*n*	Mean ± SD	Min	Max	*p*
	Age at onset (years)		74	6.79 ± 1.18	3.00	8.00		419	6.66 ± 1.38	0.10	8.0	0.335
		≤6	18				24.3					
Initial evaluation	Age (years)		74	7.50 ± 1.28	3.50	9.50		419	7.56 ± 1.47	0.75	10.58	0.479
	BMI (kg/m2)		74	17.40 ± 1.97	14.73	23.84		410	17.48 ± 2.20	12.21	25.45	0.673
	BMI (SDS)		74	0.1 ± 1.40	−2.00	4.60		410	1.29 ± 1.59	−2.4	6.6	0.958
		≥2	7				9.4					
	Growth rate the year before (cm)		70	7.55 ± 1.57	5.00	13.00		374	7.80 ± 2.17	2.00	24.00	0.593
	Growth rate the year before (SDS)		70	1.89 ± 1.80	−1.1	6.6		374	2.32 ± 2.38	−4.67	12.94	0.489
		≥2	32				45.7					
	Height (cm)		74	130.46 ± 9.57	101	146.5		419	130.63 ± 11.59	68.0	155.0	0.521
	Height (SDS)		74	2.06 ± 1.43	−1.31	6.18		419	2.1 ± 1.3	−2.9	5.3	0.913
	Bone age (years)		73	8.7 ± 1.6	4	11						
	Difference between bone and chronological ages (years)		73	1.23 ± 1.25	−0.90	5.50		405	1.3 ± 1.3	−2.5	6	0.787
		≥2	24				32.9					
	Uterus length (mm)		47	35.03 ± 8.02	21.00	58.00		223	36.19 ± 8.97	16.00	75.00	0.463
		≥35	23				48.9					
	Basal LH concentration (IU/L)		65	0.67 ± 0.83	0.10	5.00		334	0.84 ± 1.58	0.00	20.20	0.341
	Basal FSH concentration (IU/L)		64	2.04 ± 1.63	0.40	7.00		335	2.95 ± 2.09	0.20	10.00	**0.001**
	LH peak (IU/L)		74	8.95 ± 10.55	0.60	46.00		419	12.16 ± 14.90	0.20	101.0	0.059
	FSH peak (IU/L)		74	11.40 ± 5.96	0.80	36.50		419	12.84 ± 6.96	1.10	62.00	**0.045**
	LH/FSH peak ratio		74	0.96 ± 1.35	0.09	7.62		419	0.95 ± 1.07	0.04	9.83	0.170
		≥0.66	27				36.5					
	Estradiol (pg/mL)		74	13.4 ± 14.45	2.00	88.00						
		≥15	20				27					
	BAP (μg/L)		62	78.5 ± 30.3	32.1	167.2						
	CTX (pg/L)		69	5540 ± 6438.9	682	54,627						
Untreated patients			49				66.2					
	Growth rate 6 months following initial evaluation (cm)		28	3.8 ± 1.1	2	6						
	Growth rate 6 months following initial evaluation (SDS)		28	2 ± 2.5	−2.25	8						
	Growth rate the year following initial evaluation (cm)		16	7.6 ± 1.8	4.7	11						
	Growth rate the year following initial evaluation (SDS)		16	2.1 ± 2.2	−1.5	6.6						
		≥2	8				50					
	Adult height (cm)		21	163.7 ± 6.4	151	178						
	Adult height (SDS)		21	0.1 ± 1.1	−2.2	2.6						
	Difference between target and adult heights (cm)		19	−1.2 ± 3.7	−7	4.5						
		≥5	0				0					

Values are presented as mean ± standard deviation, minimum and maximum. *P*-values were calculated with Mann Whitney *U* test

*BMI* body mass index, *SDS* standard deviation score, *LH* luteinizing hormone, *FSH* follicle stimulating hormone, *BAP* bone alkaline phosphatase, *CTX* C-terminal telopeptide of type I collagen crosslinks, *n* number of patients analyzed, *MWU* Mann Whitney *U* test

Bold data correspond to significant *p* values

In all patients, CPP was diagnosed based on the appearance of breast development before the age of 8 years, accompanied by the presence of pubic or axillary hair (*n* = 47/74; 63.5%), a growth rate greater than 2 standard deviations (SDS) the year before the initial evaluation (*n* = 32/70; 45.7%), and/or BA more than 2 years greater than their chronological age (*n* = 24/73; 32.9%). (Table 1). We also considered CPP, but not premature thelarche, for girls for whom breast development at initial evaluation was clinically isolated (*n* = 13/74; 17.6%) but associated with a uterus length greater than 35 mm on ultrasound (*n* = 4/9 evaluated), a luteinizing hormone (LH)/follicle stimulating hormone (FSH) peak ratio greater than 0.66 by a GnRH test (*n* = 7/13), and/or a serum estradiol concentration greater than 15 pg/mL (*n* = 6/13). According to these criteria, only 3 girls (4%) had isolated breast development at initial evaluation, but their clinical picture became complete before 8 years of age, with ages at first menstruation of 10 and 11.5 years in the 2 patients for whom this information is available.

### Methods

Organic intracranial lesions were excluded by neuroradiological evaluation except in 12 cases (16.2%) with a familial history of early puberty, age greater than 6 years at onset of puberty, normal neurological evaluation, prepubertal serum estradiol concentrations and LH/FSH peak ratio < 0.66.

The patients were assigned to one of two groups: treated patients (*n* = 25/74, 33.8%) had been given a GnRH analog, whereas untreated patients (*n* = 49/74, 66.2%) were followed without treatment. The criteria for treatment were a predicted AH < 155 cm at initial evaluation (*n* = 4), an LH/FSH peak ratio > 0.66 (*n* = 17), and/or a serum estradiol concentration > 15 pg/mL (*n* = 8). Treatment (D-Trp-GnRH, 3.75 mg, i.m., every 24–26 days; half doses in patients weighing < 20 kg) was continued for at least 2 years. Among the untreated CPP patients, growth rate at 6 months and one year after initial evaluation were available for 57.1% (*n* = 28/49) and 32.6% (*n* = 16/49), respectively. AH was available for 42.8% (*n* = 21/49) of these patients.

The following data were collected at the initial evaluation: age at onset of puberty (corresponding to the age at breast development) and at initial evaluation; pubertal stage; height; body mass index (weight in kg/height in m^2^); growth rate the year before evaluation; BA; and evaluation of the hypothalamic-pituitary-ovarian axis by measuring basal and GnRH-stimulated LH and FSH peaks and the serum concentration of estradiol. A pelvic ultrasound was performed in 47 patients. The ensuing examinations included height and growth rate at 6 months and 1 year in the untreated girls.

Height (measured in all by R.Brauner with a Harpenden stadiometer), growth rate and body mass index are expressed as SDS for chronological age [20, 21]. The pubertal stage was rated according to *Marshall and Tanner* [22]. BA was assessed by R. Brauner for all patients according to the *Greulich and Pyle* method [23]. Target height was calculated based on parental heights reported by the parents [24].

LH and FSH concentrations were measured using a two-site monoclonal immunoradiometric assay (LH-Coatria and FSH-Coatria; bioMerieux, SA, Marcy-l’Etoile, France). Estradiol was extracted with ether and measured by radioimmunoassay (Estradiol-2; Sorin Biomedica, Antony, France). BAP and CTX concentrations were assessed by means of chemiluminescent immunoassays using the Cobas automated platform (Roche Diagnostic, Meylan, France).

For the GnRH stimulation test, we used Relefact (100 μg/m^2^) with serum samples collected at 0, 30, 60 and 90 min after the injection. Serum LH, FSH and estradiol concentrations were measured using various radioimmunoassays during the study period. Each new assay for a given hormone was cross-correlated with the previous method to ensure comparable results for a given parameter throughout the study period. The following values were considered to be pubertal: uterus length > 35 mm, LH/FSH peak ratio after GnRH test > 0.66, and serum estradiol concentration > 15 pg/mL (55 pmol/L).

### Statistical analysis

The numeric data are expressed as the mean ± standard deviation. Differences in auxological and clinical data between groups were analyzed using the Mann Whitney *U* test. Correlations were assessed with Spearman’s using GraphPad Prism (GraphPad Software Inc., San Diego, CA, USA). The magnitudes of correlation are expressed as trivial (*r* < 0.1), small (0.1 < *r* < 0.3), moderate (0.3 < *r* < 0.5), large (0.5 < *r* < 0.7), very large (0.7 < *r* < 0.9), and nearly perfect (*r* > 0.9) [25]. A *p* value < 0.05 was considered significant.

## Results

### Characteristics at initial evaluation (Table 1)

According to Tanner breast stage development, 62% of patients were stage 2 (*n* = 46/74), 36.5% were stage 3 (*n* = 27/74) and 1.5% were stage 4 (*n* = 1/74). Breast development was associated with 1 additional pubertal sign (*n* = 20/74; 27%), 2 additional pubertal signs (*n* = 21/74; 28.4%), 3 additional pubertal signs (*n* = 16/74; 21.6%), 4 additional pubertal signs (*n* = 9/74; 12.2%) or 5 additional pubertal signs (n = 4/74; 5.4%) (See Participants). One patient (1.5%) exhibited all the pubertal signs evaluated, whereas 3 (4%) had isolated breast development.

### Correlations (Table 2)

**Fig. 1:**
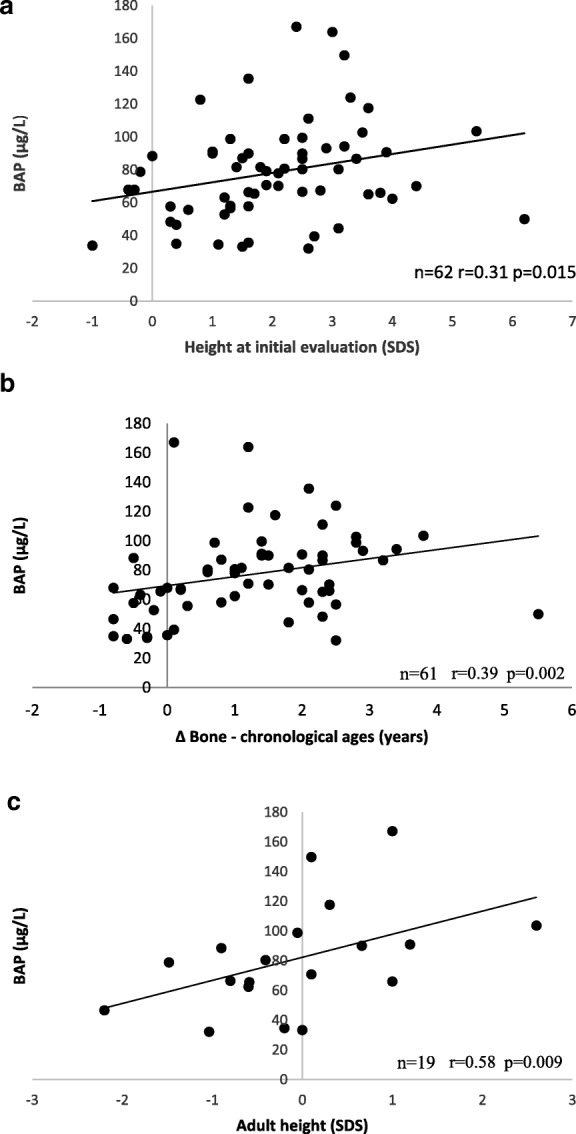
Correlation between serum BAP concentrations and height at initial evaluation (**a**), BAP and differences between bone and chronological ages in girls with CPP (**b**), and with adult height in girls with untreated CPP (**c**)

The serum BAP concentration showed moderate significant positive correlations with height in SDS at initial evaluation (*n* = 62; *r* = 0.31; *p* = 0.015) and with the difference between bone and chronological ages (*n* = 61; *r* = 0.39; *p* = 0.002) and a small positive correlation with the serum basal FSH concentration (*n* = 57; *r* = 0.27; *p* = 0.042) (Fig. 1).

**Table 2:** Correlations between BAP and CTX and characteristics of girls with CPP

		BAP				CTX			
		n	r	IC 95%	*p*	*n*	r	IC 95%	*p*
All patients	Age at onset of puberty (years)	62	−0.15	−0.40; 0.11	0.232	69	0.05	−0.2; 0.29	0.711
	Age at initial evaluation (years)	62	−0.17	− 0.41; 0.09	0.180	69	0.01	−0.23; 0.25	0.941
	BMI (SDS)	62	0.04	−0.22; 0.29	0.752	69	−0.04	−0.28; 0.21	0.761
	Growth rate the year before initial evaluation (cm)	60	0.21	−0.05; 0.45	0.100	65	0.26	0.01; 0.48	**0.035**
	Growth rate the year before initial evaluation (SDS)	60	0.10	−0.17; 0.35	0.446	65	0.34	0.09; 0.54	**0.006**
	Height at initial evaluation (cm)	62	0,10	−0.16; 0.35	0.424	69	0.09	−0.16; .32	0.480
	Height at initial evaluation (SDS)	62	0.31	0.06; 0.52	**0.015**	69	0.21	−0.03; 0.43	0.078
	Bone age (years)	61	0.19	−0.08; 0.42	0.151	68	0.07	−0.18; 0.31	0.566
	Difference between bone and chronological ages (years)	61	0.39	0.15; 0.59	**0.002**	68	0.07	−0.18; 0.31	0.566
	BAP (μg/L)					57	0.19	−0.08; 0.44	0.153
	Basal LH concentration (IU/L)	58	0.07	−0.20; 0.33	0.606	60	0.15	−0.11; 0.4	0.243
	Basal FSH concentration (IU/L)	57	0.27	0.00; 0.50	**0.042**	59	−0.02	−0.28; 0.24	0.860
	LH peak (IU/L)	62	0.24	−0.02; 0.47	0.063	69	0.12	−0.13; 0.35	0.346
	FSH peak (IU/L)	62	0.10	−0.16; 0.35	0.448	69	0.01	−0.23; 0.25	0.935
	LH/FSH peak ratio	62	0.14	−0.12; 0.39	0.263	69	0.10	−0.15; 0.33	0.415
	Estradiol (pg/mL)	62	0.16	−0.10; 0.40	0.206	69	−0.06	−0.3; 0.19	0.618
Untreated patients	Growth rate 6 months following initial evaluation (SDS)	25	0.17	−0.25; 0.54	0.406	24	−0.02	−0.43; 0.40	0.928
	Growth rate the year following initial evaluation (SDS)	13	0.31	−0.31; 0.74	0.306	13	−0.18	−0.67; 0.43	0.566
	Adult height (cm)	19	0.58	0.16; 0.82	**0.009**	17	0.04	−0.46; 0.52	0.874
	Adult height (SDS)	19	0.58	0.15; 0.82	**0.009**	17	0.04	−0.46; 0.53	0.869
	Difference between target and adult heights (cm)	17	−0.19	−0.63; 0.33	0.453	15	−0.23	− 0.67; 0.33	0.404

Serum BAP concentrations were available for 62 patients including 42 untreated patients

Serum CTX concentrations were available for 69 patients including 44 untreated patients

*BAP* bone alkaline phosphatase, *CTX* C-terminal telopeptide of type I collagen crosslinks, *BMI* body mass index, *SDS* standard deviation score, *LH* luteinizing hormone, *FSH* follicle stimulating hormone

Bold data correspond to significant *p* values

**Fig. 2:**
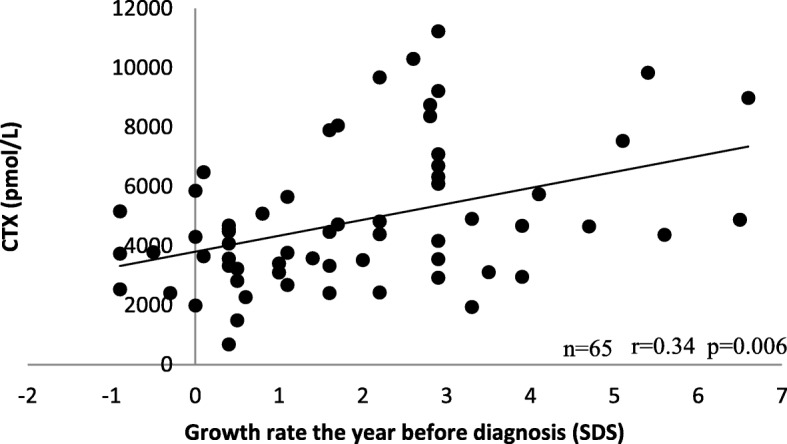
Correlation between CTX and growth rate the year before initial evaluation in girls with CPP. An extreme value of CTX (54,627 pmol/L) was removed for better visibility

The serum CTX concentration showed a small significant positive correlation with growth rate in cm (*n* = 65; *r* = 0.26; *p* = 0.035) and a moderate correlation with growth rate in SDS (n = 65; *r* = 0.34; *p* = 0.006) the year before the initial evaluation (Fig. 2).

There was no correlation between BAP and CTX concentrations. Furthermore, there were no correlations of serum BAP or CTX concentrations with age at onset of puberty, age at initial evaluation, body mass index, BA, basal LH concentration, FSH or LH peak, LH/FSH ratio, or estradiol concentration.

Among the untreated patients, there was no correlation of BAP or CTX concentrations with growth rate at 6 months or 1 year (in cm and SDS) after diagnosis.

The mean AH in untreated patients was 163.7 ± 6.4 cm. Height at the initial evaluation and AH were largely positively correlated (*n* = 21; *r* = 0.58; *p* = 0.005). The BAP concentration was largely positively correlated with AH in cm and in SDS (*n* = 19; r = 0.58; *p* = 0.009 for both) in untreated patients (Fig. 1).

## Discussion

The objective of the present study was to evaluate the relationship between the serum concentrations of BAP and CTX and the growth rate and AH in girls with CPP. BAP displayed significant positive correlations with the following parameters at initial evaluation: height in SDS, difference between bone and chronological ages, and serum basal FSH concentration. Moreover, serum BAP was positively correlated with AH in untreated patients who had spontaneous growth. The CTX concentration displayed a significant positive correlation with growth rate the year before the initial evaluation.

### Serum BAP concentration in girls with CPP

Based on the results of this study, serum BAP can serve as a marker of growth and bone maturation. A positive correlation between BAP and height was described in some previous studies [6, 26] but was not confirmed by other studies [4, 7, 27]. However, these former studies differ from the present study by several criteria, including different technical assessments of BAP and CTX concentrations (use a wheat germ agglutinin assay) [4], different studied populations (obese patients and post-pubertal patients) [4, 7], and different comparisons of bone markers to height (in cm but not in SDS) [4].

CPP can reduce growth potential because premature secretion of estradiol accelerates BA progression, followed by a reduction in growing period and subsequently in growth potential. In the present study, 32.9% of patients encountered a significant BA advance (a difference of greater than 2 years between bone and chronological ages). The positive correlation between the BAP level and BA advance suggests that BAP concentrations give an approach of epiphyseal maturation and may be useful in predicting the AH.

We found the BAP concentration to be positively correlated with AH in untreated patients with CPP. No study to date has evaluated such a correlation. The fact that BAP was correlated with height at initial evaluation, BA advance and AH is consistent with the tables proposed by Bayley and Pinneau for predicting AH, which were based on height at evaluation associated with BA at the time of assessment [28]. Specific tables were established in patients with advanced BA, and although this method is currently controversial because it is considered to overestimate AH, it has been used for decades.

Moreover, the fact that BAP was correlated with both height at initial evaluation and AH is consistent with previous studies showing that height at treatment initiation in girls with CPP is the most important positive factor influencing AH [29, 30]. This finding is reinforced by the fact that we found a large correlation between height at initial evaluation and AH. Recently designed mathematical models predict AH in patients with CPP [19] by using mother and father heights and the following data at initial evaluation: chronological age, height, and LH/FSH peak ratio. The correlation between BAP and AH suggests that it can be used as an additional tool to predict AH, potentially assisting with the decision of whether to introduce treatment with a GnRH analog. This has to be evaluated.

BAP was positively correlated with basal FSH concentrations but not with the LH/FSH peak ratio. A link between serum BAP and FSH concentrations has not yet been reported.

### Serum CTX concentration in girls with CPP

The serum CTX concentration was positively correlated with growth rate the year before the initial evaluation. This result is consistent with several studies demonstrating the link between growth rate and bone markers more specifically during puberty, in children with a normal age of puberty onset [2, 4, 6]. Growth rates and serum bone markers concentrations (BAP, alkaline phosphatase, osteocalcin, procollagen type I propeptide, type I carboxyterminal telopeptide) increase significantly in puberty compared to the prepubertal period, reaching a peak at Tanner breast stage 3, after which they decrease to reach the adult concentration just as the growth rate slows. No study thus far has evaluated the correlation between bone markers and growth rate in children with CPP.

### Strengths and limitations

A strength of our study is that we performed a homogeneous and comparable evaluation of all patients. Except for the classification of the pubertal stage, the evaluation criteria were all objective (quantitative). However, the evaluation of all patients by the same hospital practitioner allows for homogenization of this partially subjective result. In addition, as bone remodeling markers are correlated with the acquisition of bone mass in girls during puberty [4], the evaluation of these markers may allow for optimal treatment of patients whose bone mass is insufficient.

This study has limitations. It is a retrospective study, but the large number of patients and the use of a single investigator limited this bias. Although AH after spontaneous growth was available for only 21 (42.8% of the untreated) of the patients, the correlation is large. The exclusion of girls due to the absence of an available blood sample may have introduced bias. We postulate that the similarity among the data from these girls to the patients who were included, with regard to the variables analyzed (except for FSH basal and peak concentrations), limits this bias. This study did not have a control population of the same pubertal stage. The AH of the parents weren’t measured but reported which may have introduce bias [31].

## Conclusions

In conclusion*,* this study showed a significant correlation between serum BAP concentrations and AH in untreated girls with idiopathic CPP. The results also revealed several correlations with precocious pubertal features. Therefore, these bone markers could be possibly used to optimize models of AH prediction in girls with CPP.

## Abbreviations

AH: adult height
BA: bone age
BAP: bone alkaline phosphatase
CPP: central precocious puberty
CTX: C-terminal telopeptide of type I collagen crosslinks
FSH: follicle stimulating hormone
GnRH: gonadotropin-releasing hormone
LH: luteinizing hormone
SDS: standard deviation score
